# What motivates primary care providers to prescribe mifepristone medication abortion? Results of a qualitative investigation in Canada

**DOI:** 10.1186/s12875-025-03043-1

**Published:** 2025-11-10

**Authors:** Sarah Munro, Madeleine Ennis, Kate Wahl, Aleyah Williams

**Affiliations:** 1 Department of Health Systems and Population Health, School of Public Health, University of Washington, Seattle, USA; 2 Department of Obstetrics and Gynaecology, Faculty of Medicine, University of British Columbia, Vancouver, Canada

**Keywords:** Mifepristone, Medication abortion, Canada, Pregnancy, Primary care, Qualitative research

## Abstract

**Background:**

Mifepristone-misoprostol, the gold standard medication abortion drug regimen, became available in Canada in 2017. However, there is limited evidence regarding the factors that influence primary care providers to begin prescribing medication abortion. We aimed to explore perspectives of the behavioural, social, and system factors that influence implementation of medication abortion prescribing among primary care providers in Canada.

**Methods:**

We led a qualitative investigation involving one-on-one interviews with primary care providers who were interested in becoming or already were low-volume medication abortion prescribers in Canada. We collected data at two time points: (1) in 2018 after the first year of mifepristone’s availability and (2) in 2023. We recruited participants through partner health organizations’ online platforms and listservs. We conducted reflexive thematic analysis to understand resolved, novel, and ongoing factors influencing the implementation of mifepristone in primary care and mapped our results to Diffusion of Innovation theory.

**Results:**

We completed 18 interviews with primary care providers from across Canada. We identified 5 core Diffusion of Innovation factors that were important to primary care provider implementation of medication abortion care. These factors included *adoption and assimilation* (motivation), where prescriber pro-choice attitudes and commitment to provide abortion as part of generalist primary care were facilitators. *The innovation* (knowledge required to use it) and *implementation* (external collaboration) were interrelated constructs: after training in the knowledge and skills to offer medication abortion, prescribers needed ongoing collaboration and support with physician and pharmacist peers. *System antecedents* (a receptive context for change) included challenges with abortion-related stigma and harassment in professional and community settings. Finally, *system readiness* (dedicated time and resources) was necessary to ensure ease in the logistics of medication abortion care, including billing, counseling, and delays in timely care.

**Conclusions:**

Our results highlight that, after five years, barriers still exist to providing mifepristone medication abortion in Canadian primary care. We illustrate the importance of addressing ongoing perceptions of logistical barriers to care, concerns about advertising abortion services to the community, and the need for robust mentorship and consultation pathways.

**Supplementary Information:**

The online version contains supplementary material available at 10.1186/s12875-025-03043-1.

## Introduction

First trimester induced abortions are a common and essential health care service, with over 90,000 abortions in Canada reported annually [1]. In Canada, there are no laws governing abortion care, and people have the right to access abortions [2]. In 2017, the gold standard of medication abortion, mifepristone and misoprostol, became available in Canada. Strong evidence highlights the safety, efficacy, and acceptability to clients of care of mifepristone in combination with misoprostol for first trimester abortion [3]. Prior to this, according to a 2012 national survey of abortion providers in Canada, less than 4% of all abortions were medication abortions which were carried out with a less effective off-label use of a methotrexate and misoprostol regimen [4]. The majority were procedural abortions conducted at high-volume abortion clinics in urban areas.

Mifepristone availability offers the potential to expand abortion access, if the regulations around the medication could support its dispersal via primary care. While no laws govern abortion care in Canada, drug regulator Health Canada initially specified mifepristone restrictions such as: mandatory training and registration for prescribers and pharmacists, as well as physician-only direct dispensing [3]. Evidence indicated that regulations such as these would impede safety, access, and provision of medication abortion in primary care in Canada [5]. Between 2017 and 2020, Health Canada removed these restrictive regulations making it available through normal prescribing and pharmacist dispensing pathways and expanding medication abortion prescribing authority to include nurse practitioners. Evidence highlights that mifepristone has enabled a rise in provision of medication abortion in primary care, and the opportunity for rural residents to access abortion care closer to home [6–8]. The COVID-19 pandemic further catalyzed changes to medication abortion access in Canada starting in 2020, with the introduction of practice guidelines for low- to no-touch telemedicine abortion [9, 10]. 

Analysis of surveillance data suggests that new abortion providers in Canada include primary care providers who offer abortion as a part of their overall practice, with a large proportion practicing in rural communities [11]. Since the outset of the COVID-19 pandemic, the Society of Obstetricians and Gynaecologists of Canada has recommended use of a low- or no-test medication abortion protocol through telemedicine, where the patient obtains the prescription by mail or at a local pharmacy [10]. While prior research has described the abortion workforce, there is limited evidence regarding the factors that influence or motivate primary care providers to initiate abortion care. Although thousands of primary care providers in Canada have mifepristone prescribing authority, workforce survey data indicates that barriers to implementing abortion practice can include fears of interprofessional or community stigma, perceptions of limited patient demand in primary care settings, and misperceptions regarding the need for specialized training [12–15]. 

We sought to investigate perspectives on the behavioural, social, and system factors that influence primary care providers to implement medication abortion prescribing in Canada. We also sought to compare influencing factors identified during the first year of mifepristone’s availability with those identified five years later.

## Methods

### Study design

We led a qualitative investigation involving one-on-one interviews with care providers collected at two time points to understand what factors support and motivate prescribers to initiate medication abortion practice.

The theoretical framework that guided our study was Diffusion of Innovation in Health Service Organizations described by Greenhalgh and colleagues, which includes six broad constructs representing 58 dimensions [16]. We adapted Cook and colleagues’ operationalization of the constructs for our interview guide [17]. The framework posits that implementing an innovation (e.g., medication abortion) depends on its simplicity and trialability, its benefits and advantages relative to what was previously used, and its fit with adopters’ (e.g. primary care providers’) values, needs, and tasks [16]. Implementation also depends on the abilities and willingness of the adopter, the size and readiness of their organizational settings, and the health system support and resources available [16]. 

We collected data in 2018 as part of an interview study nested in the Contraception and Abortion Research Team-Mifepristone (CART-Mife) Study. This was a national, four-year, prospective, mixed-methods program of research on factors that influenced mifepristone provision in primary care in its first year of use in Canada [6]. Second, we collected data in 2023 as part of the CART Access Project, a federally funded program of research aimed at generating evidence and tools to sustain and improve equitable access to abortion in Canada [18]. The Behavioral Research Ethics Board of the University of British Columbia and BC Women’s and Children’s Hospital provided ethical approval.

### Setting and participants

Eligible individuals included English or French-speaking primary care providers who were self-defined low volume medication abortion providers or who were interested in prescribing medication abortion but had not yet initiated the practice in a Canadian health care setting. In Canada, eligible prescribers include physicians, nurse practitioners, and in some settings midwives. We excluded individuals who were ineligible to prescribe mifepristone. We restricted settings to any service delivery environment where a prescriber could provide medication abortion in primary care, including hospitals, sexual health clinics, abortion clinics, health centers, and private physician offices, and via in-person care or telemedicine. We engaged in purposeful recruitment to ensure participants represented a range of professions, practice settings, regions, rural/urban locations, ages, and genders.

We recruited through advertising via partner organizations whose membership included eligible individuals with a high likelihood of being eligible to participate. In 2018, we advertised via email listservs hosted by the Canadian Abortion Providers Support platform (www.caps.cpca.ubc.ca), College of Family Physicians of Canada (Maternity Care Discussion Group [MCDG]), and through “snowball” sampling. In 2023, we again advertised via email listservs hosted by the Canadian Abortion Providers Support platform and added third-party recruitment via social media posts and email listservs for nurse practitioners and newly graduated family physicians, gynaecologists, and pediatricians specializing in adolescent medicine. Interested individuals reviewed the consent form ahead of time to ask questions, and provided written or verbal consent prior to the interview.

### Data collection

We audio-recorded interviews by telephone or Zoom. Two family physician residents conducted the 2018 interviews, while a researcher with public health training (AW) completed the 2023 interviews. The interviewers received mentorship from an experienced qualitative researcher (SM) to ensure methodological rigour and consistency of approach. The interview began by collecting basic demographic information, followed by flexible open-ended questions to explore the barriers, challenges, and opportunities for implementation of mifepristone in primary care (see Supplementary Appendix A, Interview Guide) [6], with questions and phrasing guided by Greenhalgh’s framework for Diffusion of Innovation Theory [16]. In our initial interviews we sought to understand key implementation factors at mifepristone’s initiation. After five years, we explored ongoing challenges and necessary supports for prescribers to initiate and sustain medication abortion in primary care, in particular for providers with a low volume of cases (see Supplementary Appendix B, Interview Guide). At both time points, we continued sampling until we had satisfied key markers of saturation: additional interviews yielded data that were consistent with previous data; no new themes were identified in analysis; and each theme was demonstrable within the sample [19, 20]. As a team we discussed the sample, our memos, and initial analysis before closing recruitment at each time point.

### Analysis

We transcribed, de-identified, and assigned a non-identifying numeric identifier (e.g., P001-year) to the audio-recorded interviews. Three qualitative abortion researchers (SM, AW, ME) conducted iterative rounds of analysis across the study period. We selected a reflexive thematic analysis approach as it centres researcher subjectivity and an awareness that knowledge is constructed, situated, and contextual [21, 22]. The steps included data familiarization (reading and re-reading the transcripts and field notes, making notes about analytic ideas), coding (systematic identification of data that captures a meaningful concept), generation of initial themes (compiling codes that represent a core idea that could meaningfully address the research questions), refining, defining, and naming themes, and writing up (building the analytic narrative about how the dataset addresses the research questions). Finally, we deductively mapped the results of this analysis (codes, patterns, and relationships) to the Diffusion of Innovation framework [16] through iterative team-based workshopping sessions and during manuscript preparation. We resolved any discrepancies through discussion and reaching consensus among the study team.

## Results

We completed 18 interviews with primary care providers. We detail participant demographics in Table 1. Eight were family physicians interviewed in 2018 who had not yet prescribed mifepristone medication abortion. Four of the eight participants practiced in rural regions, with practice settings including medical offices, community clinics, urgent care, and hospitals. The 10 participants interviewed in 2023 included five family physicians, four nurse practitioners, and an adolescent medicine specialist. Eight of these 10 participants were prescribing mifepristone medication abortion at the time of the interview, each with less than ten abortion cases per year. They spoke to experiences of practice and training across the country, including in Atlantic, Central, and Western Canada.

**Table 1:** Characteristics of the sample (*n* = 18)

Characteristic, *n* (%)	2018 participants (*n* = 8), n (%)	2023 participants (*n* = 10), n (%)
Gender		
Man	3 (38)	2 (20)
Woman	5 (62)	8 (80)
Age		
<30	0	0
30–39	8 (100)	5 (50)
40+	0	5 (50)
Rural^1^	4 (50)	5 (50)
Urban^1^	4 (50)	5 (50)
Discipline		
Family Medicine	8 (100)	5 (50)
Nurse Practitioner	0	4 (40)
Adolescent Medicine Specialist	0	1 (10)
Previous abortion experience^2^		
Yes	4 (50)	8 (80)
No	4 (50)	2 (20)

^1^We defined urban participants as those located within Statistics Canada’s defined census metropolitan areas (CMA), with all other participants classified as rural

^2^Among those who had provided abortions before mifepristone’s availability, their experiences were diverse and ranged from writing one prescription for methotrexate-misoprostol to full-time procedural abortion practice to previous hands-on training during school or residency

**Fig. 1:**
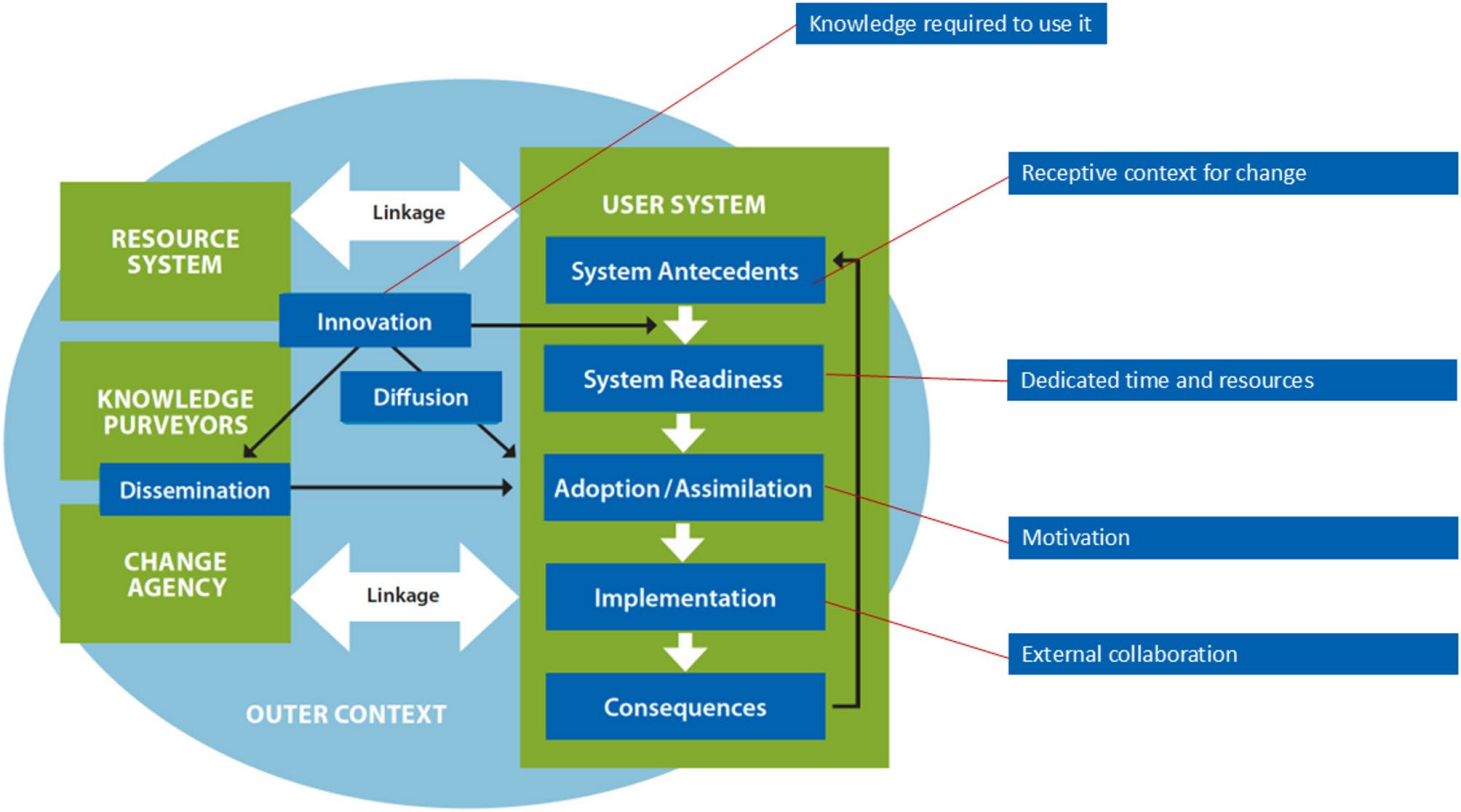
Determinants of diffusion of medication abortion among primary care prescribers, adapted from Greenhalgh et al. [16] Source: Norman et al. [23]

We identified 5 core Diffusion of Innovation factors that were important to primary care provider implementation of medication abortion care, and mapped relationships between them (see Fig. 1). These factors included *adoption and assimilation* (motivation), where prescriber pro-choice attitudes and commitment to provide abortion as part of generalist primary care were facilitators. *The innovation* (knowledge required to use it) and *implementation* (external collaboration) were interrelated constructs: after training in the knowledge and skills to offer medication abortion, prescribers needed ongoing collaboration and support with physician and pharmacist peers. *System antecedents* (a receptive context for change) included challenges with abortion-related stigma and harassment in professional and community settings. Finally, *system readiness* (dedicated time and resources) was necessary to ensure ease in the logistics of medication abortion care, including billing, counseling, and delays in timely care. We provide our coding framework and additional representative quotes in Table 2.

**Table 2:** Results of the thematic analysis

Theme	Sub Theme	Year	Example Quotes
Adoption and assimilation: Motivation to prescribe	Commitment to provide abortion	2018 & 2023	“I feel like it should definitely be accessible. I feel like it’s the choice of the person who is going through it. I recognize it’s a hard decision for someone. It’s a challenging topic, but overall, I feel like yes, it is something that needs to be accessible to any woman or individual who wishes to access it” (P041–2018) “I support women’s choice” (P044-2018) “I think that’s part of the reason I wanted to partake in the study. It was actually to learn a little bit more. Something that I think if I had a bit more information about or if I could develop something here, that I would be happy to work on and to champion or at least communicate with my fellow colleagues here.” (P038-2018) “The more we can normalize it and integrate it in primary care, the better for patients.” (P103-2023) “Once termination is decided on, I talk about it like any other medical procedure” (P102-2023) “Medical abortion is in the wheelhouse of general practice, and I take pride to provide this service to my patients. It makes it one step simpler for patients who can access it without referral” (P102-2023) “I would have a hard time working in a clinic that made me feel like I couldn’t provide abortion care or questioned my decision to prescribe or if a doctor part of the clinic told their patient ‘no’” (P110-2023)
	Referring patients to high volume clinics due to perceptions of ease and low demand	2018 & 2023	“It’s just easier from a patient perspective and from a physician-monitoring perspective to have them go to this clinic where it’s all tied up in one package.” (P038-2018) “Because we are so close to [big city], that is mostly where I go. I know that occasionally terminations, surgical terminations I think maybe done at my hospital. I heard of one, but I don’t know if it’s a routine thing, so I refer to [big city], the clinics there.” (P044-2018) “I’m lucky enough to be working in a part of the city where we have very easy access to a high-volume abortion care provider, so I make a quick referral, and it’s easier for women to get in and have that service provided.” (P042-2018) “I have a bit of like background education and so I feel fairly comfortable kind of counselling them about the options and then normally I would, if they did decide they wanted to pursue an abortion, then I would refer them to one of our local sexual health clinics that I know offers that.” (P109-2023)
The Innovation: Knowledge required to use it	/	2018 & 2023	“I think one of the biggest barriers is the fear of the unknown, and learning about it is a good thing even if you learn about it and choose not to do it.” (P037-2018) “Somebody advertise training. I just need to be prompted for everything because I’m pretty busy… I think for a lot of us it’s just advertising and getting in our face a little bit because life is busy.” (P40-2018) “Yeah I did the SOGC course; it was like 6 modules. I just did it online. Found a fairly good kind of algorithm to follow, so every time somebody presents, I just sort of haul that out and it’s got, you know, the blood work and the contraindications and everything is just listed there. It changed a bit with the pandemic too, so the no touch abortion that they talk about is certainly possible. It’s much more accessible to do it through phone.” (P105-2023)
Implementation: External collaboration	/	2018 & 2023	“I definitely want to have a working relationship with someone who will make it, at least when I start out, more comfortable… and kind of knows the pros and pitfalls, and someone I can get a hold of.” (P042-2018) “If the woman has a prescription for Mifegymiso and then hemorrhages, I’m curious what their [an OBGYN’s] reaction would be when I’m calling them to say, ‘I need your help. This lady needs an emergency D&C.’” (P039-2018) “Possibly a relationship [with] the main pharmacies so that I know that my prescriptions are going to be [approved], that my patients are going to be treated properly.” (P038-2018) “I mean the other [health care providers] and I support each other for all kinds of different things, so that’s very helpful. But again, I was the only person offering this care, so as things have come up, I have reached out to another colleague who I know is providing this care or I’ve had really good, always gotten really good communication back from [mentor] if I call there and I’m like oh I have a clinical question in providing this care, they’re always like super supportive so that definitely makes me more confident and comfortable especially because I do the care infrequently relative to the other providers. You know, and then also to in terms of next steps if things don’t go well with the abortion and they do need like a next step, how to get them connected and how best to do that, so yeah, I find that’s really helpful” (P103-2023)
System antecedents: A receptive context for change	Experiencing anti-abortion stigma	2018 & 2023	“We do have a couple of administrators in our [health authority], one in particular who is not cool … when it [abortion] is surgical based, who is personally and religiously against abortion. He’s been very obstructive, and he’s really kind of kiboshed having a regular abortion clinic here, because everyone else is completely willing and working to make that happen. In [rural town], the administrative medical staff are, again, morally or religiously opposed and tend to really put up barriers to that happening here.” (P40-2018) “The other way that colleagues have impacted our practice here locally is we have a group of very anti-choice radiologists at our local hospital, which is our main diagnostic imaging facility, who actually will not read scans if they are for the purpose of termination so that has been very challenging since we have to send people to the next town over in order to access dating scans. Does not comply with college regulations, but that’s a problem for another day.” (P107-2023)
	Hesitation to advertise	2023	“I have never advertised that I provide MA other than internally and with my colleague. If you’re a patient and you google abortion in [redacted], you won’t find any providers and that’s 100% driven by my community and the fact that I have a young family. The previous provider who did it for 40 years did advertise and had red paint thrown on him. I don’t hide it, if patients and colleagues ask, but it’s not an advertised service” (P107-2023) “All my friends know I provide this service. Right now, I’m challenged because I share a physical office with five other physicians who are not pro choice, one is staunchly anti choice so I would need a separate office that’s my own. I live in a town where people stand on a main street corner with anti choice signs every Sunday year-round. There is a big church based anti choice lobby, to the point when I was thinking about writing a letter to radiologists, that they would share the letter with the church and everyone knows where I live. I’m not afraid of violence but I’m definitely afraid of the ramifications for my kids and husband” (P107-2023) “Having a rainbow flag or something like it in our waiting room would be interesting because we share it with the birthing program. I think it would be good. I would be interested to hear what the clinic would say to that. It shouldn’t be something that we have to hide. if it was just our clinic, it would be easier, but it would have to be a discussion between two clinics because we share a waiting room. but i think we should because we do offer that care and it would save patients from having to stress and look around; they could just come to us.” (P110-2023) “Rainbow flags… tricky because we advertise it as a safe space but then not everyone is on board. I have flags in my exam room. This would be tricky to advertise though because if you put a notice on the website or in the waiting room, its polarizing and you’ll get messages from people who question whether its ethical or safe. could create safety issues for anyone in the office who may not be careful taking that risk. which is different than when you work in a specialty and people know about that risk.” (P103-2023) “Having a secure list would be a good idea. Put people into categories based on how they’re accessed. Because I’m only available to women’s college family practice patients. Including open access providers would be good” (P103-2023). “It would be nice to have a way for patients to ask for MA without asking their primary provider, similar to how requests for MAID are done through a referral centre” (P102-2023) “Secure list? Absolutely. As a provider you feel like you or your family could be targeted at some point, but it is important for providers to know who they can refer their patients to versus having them sent out the door with resources…I trust my college to make a list secure but then it wouldn’t be comprehensive because it would only include NPs. So it would have to include things like an organization who’s very tech savvy, so they won’t be hacked, and who will have access to that list?” (P106-2023)
System readiness: Dedicated time and resources	Awareness of and access to remuneration for prescribers	2018 & 2023	“It’s more billing codes and people not prescribing because they don’t feel like they’re paid for the time.” (P41-2018) “I don’t think there’s any special billing codes for that, just normal family practice. I’m not doing it because I think it’s a financial gain, I do it because I feel it’s a needed service… If I was doing it 4 to 5 times a week and getting 10 to 12 phone calls every night and being the only one responding to these emergency phone calls then it would be a lot and it wouldn’t be sustainable but for now it’s a telehealth phone call and we still get paid” (P110-2023) “The actual medical abortion provision code is out of basket and it’s only billable if a patient is seen in person. A large number of the abortions I’ve done were in 2021 and billing then was a real challenge in so much as we barely got paid for most of that provision. If it was my own patient, I was making $3 and if it was my colleague’s patient, I would get $30. I view it was volunteer work. This may have changed because you can now get the same fee for phone consults as in person visits less 15%” (P107-2023). “The physicians are overwhelmed in primary care and feel like there are extra skills that would take time away from it and maybe have some discomfort around managing complications. Even if there was conscientious objection in the context of a miscarriage, they will usually refer to an early loss clinic. I have one colleague who’s expressed interest in taking that on and managing it themselves, but I don’t think they’ll do it because they’re still overwhelmed especially post pandemic. It feels like one extra thing they have to offer. I’m not aware of anything overt other than the time management issue” (P103-2023).
	Having time for counseling and follow-up	2018 & 2023	“Well part of the reason why I don’t do a lot of medical abortion, because we have a lot of transient sort of people in this town, a lot of people that move around here, work the back country, work remotely, and the follow-up is a real problem. I’ve had a few people kind of flake out for their medical abortion, and it’s really scary when they don’t turn up for follow-up. You have to track them down, and it’s really hard in a fee-for-service environment.” (P040-2018) “Then the next follow up with them is checking in to see how everything is going and then the follow up; I do have a practice of doing baseline blood work and then doing repeat blood tests 7 to 10 days later. occasionally patients don’t do that, and I’m chasing them down, and then I, you know, I have to say well they’re making informed choice like I can’t do much about that. but I do try to emphasize the importance of making sure that everything is complete. I have had two instances where patients needed like further intervention, so it is important to follow that up.” (P103-2023)
	Navigating an over-burdened primary care system	2018 & 2023	“I think the biggest downside that I see here, just our challenging with ongoing monitoring. We don’t have ultrasound in our community, so they have to go out of town for that anyway. Sort of the biggest barrier for us is ongoing monitoring. One of my concerns is if they were to have heavier bleeding or anything like that, we’re not very equipped here as well. It’s mostly the ultrasound monitoring. That’s the biggest barrier I think.” (P038–2018) “Access to lab work is challenging and some patients don’t have daytime availability to go wait for a couple hours at a lab and its not something that women take time off for, probably often they could do that but they don’t because they don’t want to disclose. there’s overall a misconception when you take sick leave that you need to tell your employer why” (P108-2023) “Well, I think until quite recently we were hearing there’s only three tablets of Mifegymiso [locally]. Anyway, there was certainly some access issues there, and also, when it was just misoprostol, I guess there was lots of access to misoprostol. I guess access then wasn’t an issue. The new one has been tight until now.” (P039-2018) “In the meantime, I will also confirm that the medication is available because we’ve had issues with back orders so I’m usually calling around to make sure that I can find the medication for them. That’s been a big issue. The other thing is, we’re still really restricted in [province]. A lot of pharmacies will not offer it and so in [city], I usually have to get them to come to one of the hospital pharmacies and the interesting thing is, in rural areas, I’ve had pharmacists who are willing to order it and bring it in and give it to patients, but in the city, they like refused, which is ridiculous. so that’s kind of annoying. So I’ll usually locate the medication to make sure that they have it available, then once I know they have it available, I’ll connect with the patient again and send them lab requisitions and I send the prescriptions directly to the pharmacy, and then I follow them.” (P103-2023)

### Adoption and assimilation: motivation

#### Commitment to provide abortion

Across the two time points, participants felt motivated to offer medication abortion in reaction to perceived gaps in the healthcare system, as encapsulated in the quote, “anything I can do to kind of help improve access, I’m in.” (P105-2023). Several remarked on their ‘surprise’ and disappointment in the limited access to medication abortion, both in 2018 when it was implemented after a lengthy federal approval period, and in 2023 after being available without restrictions for five years. These motivations were rooted in participants’ belief that people have a right to choose what to do with their own bodies. One provider shared that they felt motivated to provide medication abortion to reduce access barriers: “women in this country don’t get the health care that they’ve been promised and there are all these barriers to care” (P103-2023). For others, the motivation to provide abortion in primary care reflected a desire to support and “insulate people going through abortions so they don’t experience any more trauma” (P106-2023).

In 2018, while participants had not yet initiated abortion practice, many were motivated to provide medication abortion to better serve their patients. They were excited about the potential of mifepristone to increase options and “ease” for some patients. One participant described providing abortion as an essential form of “harm reduction” (P039-2018). Participants in the 2023 sample demonstrated they were motivated by similar factors. These “early and happy adopters” (P104-2023) viewed medication abortion as part of basic primary care, reflecting “family medicine is cradle to grave” and abortion care “should be part of everyone’s family practice” (P105-2023).

#### Referring patients to high volume clinics due to perceptions of ease and low demand

Several providers in 2018 were less motivated to initiate medication abortion practice because of perceptions of ease for themselves and the patient and low patient demand. A rural GP noted: “Since we’re in such a small corner of [British Columbia], I don’t anticipate a lot of talk about it or a lot of buzz around that kind of thing.” Participants highlighted that they had strong referral pathways with other abortion providers, in particular in urban environments, leading some to feel most comfortable to continue sending patients to ‘the experts’:*“At this point*,* it’s pretty easy to refer”* (P037-2018). In 2018, existing referral pathways incentivized a ‘practice as usual’ approach whereby providers referred patients rather than provided abortion care themselves. However, among the 2023 sample rural practice was perceived as a place where abortion is particularly needed: “as a generalist in a small place, I feel it’s something you have to provide” (P108-2023). This participant went on to describe, “I’ve seen patients referred to clinics that require travel which is unfortunate when it can be provided locally” (P108-2023).

Both examples illustrate a key motivator among prescribers – patient demand. Among 2023 providers, several described having ongoing trusted relationships with their patients, for instance to provide adolescent mental health care and/or sexual health services. The trust created “a relationship based on talking about things they wouldn’t necessarily talk about with other providers” (P106-2023). These providers felt it was important they not refer their patients elsewhere and provide abortion care in a safe, trusting space.

### The innovation: knowledge required to use it

Access to skills training to have the knowledge required to prescribe medication abortion was a key factor for participants to initiate medication abortion practice. In 2018, participants were largely unaware of training for medication abortion practice in primary care. One participant (P039-2018) indicated that training is important not just for procedural skills but also for changing attitudes, reducing their fear of this new-to-Canada practice. After training options were described to them during the interview, participants were enthusiastic to participate and “have some concise information on [mifepristone]” (P038-2018). In contrast, training opportunities were ubiquitous in the follow up period, with participants citing a range of ways they accessed education: clinical practice guidelines, step-by-step protocols developed by health professional organizations, online training with Continuing Professional Development credits, in-person workshops at clinical scientific meetings. All participants described the learnings as easy and straightforward. For would-be providers who had not completed training, they cited their workload as the key barrier. Any guidance “needs to be something easy because we are so swamped these days” (P101-2023). Other training barriers included cost such as resource paywalls and lacking continuing professional development funding.

Many participants in the follow up interviews described the importance of using practice and counseling tools to streamline care. Selecting, adapting, and then using a range of point-of-care resources facilitated their low volume abortion practice. These were typically developed by colleagues or trusted health professional organizations and saved to a Dropbox or electronic medical record system for quick access. Resources included patient facing information sheets, infographics of what to expect, and how-to checklists for providers. For instance, “The [University of British Columbia] MA prescriber checklist is my go-to; that’s where I go to get all of my updates and information to know I can do something safely” (P106-2023). Participants emphasized that print resources were especially helpful to follow the steps in a medication abortion: “The simplicity of the [College of Family Physicians of Canada] one pager -- that is dead easy to follow” (P102-2023). P107-2023 shared “It is not as complicated as it is made out to be. There’s an impression among family docs that they have to do that SOGC [Society of Obstetricians and Gynaecologists course or get a special license which was the case at the beginning. There’s no special license, any family doc can do it.” Another participant shared: “It can be daunting when someone comes every two or three months, you have to remind yourself of everything but that’s why you have an information sheet at the ready” (P106-2023). Half of low-volume abortion providers continued to use a consent form to guide their conversation with patients, but none required the patient to sign it. One participant described how they adapted a form to better fit the workflow in their specific setting: “I used the hand-out from the manufacturer but added my own comments, so everything is explicit for patients and I add that to our EMR” (P103-2023).

### Implementation: external collaboration

Expert ‘back up’ was a critical facilitator for participants in both 2018 and 2023, reflecting their attitudes that medication abortion care in primary care works best when generalists and specialists are able to learn from one another. In 2018, prior to initiating practice, participants wished to secure mentorship from a supportive gynaecologist or other abortion provider, to ensure timely, trusted feedback, as highlighted by P042-2018: “A way to share experiences and kind of learn about best practices. It would also be good to have almost like a mentor to just touch base with every once in a while, potentially also to discuss more difficult cases.”

In 2023, low-volume prescribers had strong mentorship structures in place, highlighting this as a key facilitator: “Having access to the RACE line [Rapid Access to Consultative Expertise] in [British Columbia]. is super important for someone like me who doesn’t provide a lot of M.A. [medication abortion]. Knowing that I have a specialist one phone call away if something doesn’t go according to plan for my patient is huge” (P106-2023). One participant shared that they think having peer-mentors in primary care will be less challenging over time: “It is going to get easier. You won’t be a pioneer forever. The number of colleagues and peers who have experience and are available to give you advice continues to grow” (P104-2023). Participants also expressed a need to build out their interprofessional network with pharmacists, to know which pharmacies carried the medication and how pharmacists felt about dispensing mifepristone. Relationships with pharmacists were an ongoing, important need, to protect patients from being turned away by pharmacies because of anti-choice attitudes or lack of stock.

### System antecedents: a receptive context for change

#### Experiencing anti-abortion stigma

Participants displayed some concerns about whether their professional and community environment would be receptive to the prospect of abortion being offered in local primary care, primarily due to experiences of anti-abortion stigma and harassment. In one instance, a participant’s effort to implement mifepristone across their clinic team during the pandemic “was shut down” due to their colleagues’ lack of “comfort and fear with complications, liability, and generally feeling overwhelmed with other demands” (P103-2023). Another participant described a health authority administrator who had “been very obstructive” (P040-2018) and they opted not to provide abortion care to ensure an easy working relationship. One participant (P107-2023) described how radiologists at their local hospital had refused to read ultrasound scans for patients seeking abortions. Therefore, the participant offered abortion seekers two options – lie about the reason for the scan or travel to a supportive out-of-town clinic. They worried that the radiologist might retaliate by releasing their name to the local church, potentially endangering their family. Conversely, several participants were unable to think of examples of anti-abortion attitudes in their practice setting. One non-prescribing participant felt motivated to initiate abortion care because of their team’s willingness to initiate it.

#### Hesitation to Advertise

These experiences and perceptions of stigma and harassment in turn impacted participants’ motivation to implement or advertise medication abortion care, for instance online or with a sign or symbol posted in their clinic. Concerns included potentially ‘alienating’ their broader patient panel or discouraging parents from bringing in their children, in particular: “I don’t want to be known for that because it’s a small town and I don’t want to alienate a portion of my own practice who might have their own thoughts and feelings” (P102-2023). Multiple participants worked in clinics with a shared waiting room where signage would be a joint decision between owners. The participants who were actively providing a low-volume of medication abortions served their own patient panel and received referrals from nearby procedural abortion clinics and word-of-mouth.

### System readiness: dedicated time and resources

#### Awareness of and access to remuneration for prescribers

At a health system level, participants’ accounts suggested that awareness of and access to adequate remuneration for prescribing in primary care was a key implementation facilitator. Across samples, participants described the payment model and limited knowledge of abortion fee codes as leading contributors to their hesitation to provide abortion care. In 2023, many participants described abortion care as their ‘charity work.’ One physician had been part of a pilot project that partnered physicians and midwives to provide medication abortion which felt more sustainable because while they were “paid very little, most of the time-consuming tasks were done by the midwives” (P107-2023). Relatedly, for salaried nurse practitioners and physicians, remuneration was not a deciding factor: “there’s no financial disincentive to not provide the care” (P103-2023). For some physicians remunerated by fee-for-service models, higher fees set by specific provinces were incentives to provide. One participant from the Territories, for instance, shared that “the fee code for medical abortion is probably the highest that I can bill as a GP. It’s $200 to 300. Sometimes there is counselling involved which is a higher fee code” (P108-2023). In 2018 participants expressed concerns about costs for patients who did not have extended health benefits or were “marginally employed,” while conversely, no participants expressed similar concerns in 2023.

#### Having time for counseling and follow-up

Our analysis identified the role of time for counseling and follow-up as a barrier to the sustainability of medication abortion in primary care. Physicians described not having time in their fee-for-service model for fulsome counselling and medical visits. Family physicians noted the importance of interprofessional teams and reflected that support from allied health professionals such social workers or midwives would improve sustainability and prevent physician burnout. Participants perceived follow-up paradoxically as both necessary for patient safety, but also burdensome financially and psychologically, particularly where follow-up may be unpaid in a fee-for-service model. While on-call support is “a safety necessity” for medication abortion, “it’s not realistic for providers to have 24/7 availability for every patient who they provide termination to” (P107-2023). Several who were part of call groups expressed concerns about the appropriateness of on-call care provided by their colleagues who do not provide abortions. Across both time points, participants also shared concerns that *“follow-up is a real problem”* at a patient level, sharing examples of vulnerable abortion seekers who could not reliably complete a follow-up appointment within a traditional in-person model-of-care.

#### Navigating an over-burdened primary care system

For would-be providers, the time-sensitive nature of scheduling medication abortion care, and perceptions of how long it would take to support the logistics of a medication abortion, were clear deterrents to implementing practice. Patients in Canada can experience significant waiting times for primary care appointments, thus scheduling a patient for a timely medication abortion prescription may be a logistical challenge for a family doctor, even in the current context of care where guidelines support telemedicine and there is no requirement for ultrasound dating [24]. Existing providers demonstrated a commitment to ‘make time’ despite having limited resources. Several spoke of seeing patients seeking an abortion the same week or ensuring any indicated tests and prescriptions are completed before the weekend: “I made time for these visits. Some of my colleagues are months and months out. I make myself available.” (P102-2023).

Similarly, another clinician shared, “I would hate for someone to be denied a medication abortion because of logistics – because it takes too much time to pull all the pieces together” (P101-2023). Again, despite availability of no- and low-touch medication abortion protocols in Canada, participants in 2023 described ongoing diagnostic barriers to providing care, including that “access to lab work is challenging” (P108-2023) in particular for rural patients. Several described how they removed logistical barriers. One participant fast-tracked ultrasound orders while others were comfortable to routinely offer telehealth appointments or had prescriptions dispensed from their rural hospital pharmacy, where stock was more reliable and confidential than in the community pharmacy. Half of the 2023 sample invested time with each mifepristone case to find pharmacies that stock and offer day-of dispensing “because no one wants to wait if they’ve already made up their mind” (P110-2023). Overall, despite these logistical barriers within an overburdened system, participants reflected that mifepristone prescribing in primary care was safe, effective, and straightforward:

“If I could talk to my past self, I would have done it earlier in my career. I thought it was very complicated and required a special skill set but now that I’m doing it, I realize its way lower risk than many of the other medications that I comfortably prescribe everyday with way less conversation like birth control pills. There are a million things that we prescribe without batting an eye that have way higher risk. It doesn’t need to be an OB or a higher skilled provider.” (P107-2023).

## Discussion

We analyzed interviews with primary care providers from across Canada in 2018 and 2023 to understand resolved and ongoing factors that influence motivation to begin and sustain medication abortion practice. Our results suggest that while several barriers to mifepristone implementation in Canada have resolved since 2018, many barriers remained in 2023. While 2023 participants had better access to training and mentorship structures, challenges with logistical supports persisted: accessing lab work quickly and close-to-home, finding local stocks of mifepristone, providing follow-up care for patients, and navigating fee codes. Participants at both timepoints described experiences of interprofessional anti-abortion stigma and, in 2023, hesitation to advertise their abortion services.

Our results suggest that efforts by Canadian organizations such as Society of Obstetricians and Gynaecologists of Canada [25] and the National Abortion Federation [26] to offer training opportunities for medication abortion care have been successful. While participants in 2018 lacked knowledge about mifepristone and training opportunities, our 2023 sample were confident in their abilities to access training and prescribe mifepristone, often relying on step-by-step protocols. The COVID-19 pandemic catalyzed wide spread change in the Canadian abortion care landscape, including rapid uptake of telemedicine approaches [9, 24], which is supported in various training modules [25] and clinical practice guidelines [10]. Similar to other research [9, 24], our participants highlight telemedicine options as a patient facilitator to accessing abortion care.

Our 2018 sample desired mentorship from other medication abortion providers and obstetrician-gynaecologists before starting to prescribe mifepristone. By 2023, most participants had mentors and highlighted them as key facilitators of mifepristone implementation in their practices. Since 2019, the Canadian Abortion Provider Support platform has provided resources, troubleshooting, and mentorship to medication abortion providers [27]. Additionally, with the rise of mifepristone implementation in primary care settings and growth of the abortion workforce [8, 15], primary care providers are more likely to have colleagues who offer this service and provide guidance. Increasing mentorship opportunities, such as a recently implemented National Mentorship Hubs initiative, through which regional hospitals support mentorship for primary care abortion providers, may improve provider motivation to initiate abortion practice.

In 2018, participants expressed concern over cost of medication abortion services to patients. Like many other medications in Canada, mifepristone was initially self-pay at a cost of CAD $300–450. During its first few years of availability, provinces and territories introduced coverage for the drug for their residents [28], explaining why our 2023 sample did not raise this barrier.

Despite these improvements, there were several key ongoing barriers to care. Often patients are required to travel for care, including lab work such as ultrasound and blood tests. While current Canadian guidelines do not indicate testing in the absence of certain clinical factors (e.g. IUD in place during conception), previous research in Canada suggests hesitancy to forego testing [24]. In a qualitative study, physicians described that poor access to local dating ultrasounds limited their ability to provide first-trimester medication abortions [6]. Concerns about loss-to-follow up for patients navigating complex personal and system barriers to care highlights the potential to further support provision of care via telehealth or text message.

One prior study investigated experiences of implementation of medication abortion direct dispensing in Canada, also guided by Diffusion of Innovation theory [29]. In-depth interviews were completed with 24 pharmacists in 2017, among whom 33% of participants had stocked and 21% had dispensed mifepristone-misoprostol medication abortion. Thematic analysis indicated several similar constructs impacted dispensing practices. Pharmacists were motivated by the same attitudes: providing abortion care was aligned with personal pro-choice values and they perceived that medication abortion would benefit their patients. Pharmacists similarly emphasized the importance of external collaboration with prescribers. However, while pharmacists in 2017 sometimes perceived unsupportive, anti-choice attitudes among other professionals, they did not have the same degree of concern about stigma and harassment as prescribers in the present sample. Rather, provision of medication abortion was facilitated by pharmacy organizations, corporate bodies, and influential individuals who actively encouraged implementation.

Stigma from other health care professionals and community members continues to be a concern for abortion providers in Canada. A 2019 survey of Canadian abortion providers explored experiences of stigma and harassment and found that while providing abortion care in Canada is largely considered safe, especially for low volume abortion providers, it is still accompanied with perceptions of stigma from community and colleagues [14]. Similar to our results, qualitative analysis from that study highlighted that clinicians want to be in control of how and to whom they disclose their work in abortion care, and have a hesitancy to advertise their services.

In both 2018 and 2023, participants described the payment model and lack of knowledge of eligible fee codes as leading barriers to provide abortion care. This varied somewhat across participants, highlighting that abortion provision is more sustainable in provinces with higher fee codes, for salaried primary care providers, and for those with interdisciplinary practices wherein a salaried colleague could provide abortion counselling.

Our study presents several strengths and limitations. Data collection across multiple time points allowed us to identify resolved, onging, and novel barriers to medication abortion in primary care. We used a comprehensive, theory-driven interview guide to understand factors influencing the implementation of mifepristone in primary care and what supports could be helpful at its initiation. The applicability of our results is strengthened by our varied sample, which included both current mifepristone prescribers and those who had not yet prescribed. Our findings should be cautiously applied to jurisdictions where access to abortion is constrained by mandatory training, cost, and/or legal restrictions. Our results may be generalizable to settings adopting new approaches for prescribing and dispensing medication abortion in primary care and community settings, such as on college campuses and through pharmacist dispensing [30, 31]. Evidence indicates that mifepristone dispensed outside of hospitals, clinics, and medical offices is safe and acceptable to both patients and prescribers [32, 33]. 

## Conclusions

Our results highlight that barriers persist in providing mifepristone medication abortion in a primary care setting in Canada. This evidence can inform efforts to improve access to safe and high quality abortion care. We illustrate the importance of addressing ongoing perceptions of logistical barriers to care, concerns about advertising services to the community, and the need to sustain robust mentorship and consultation pathways.

## Supplementary Information

Supplementary Material 1

## Data Availability

The datasets generated and analysed during the current study are not publicly available due to individual privacy rights of our participants and as outlined to them during the consenting process.
