# Supplementary material for: What motivates primary care providers to prescribe mifepristone medication abortion? Results of a qualitative investigation in Canada
Source: BMC Prim Care. 2025 Nov 10;26:349. doi: 10.1186/s12875-025-03043-1 (PMC12599077; doi:10.1186/s12875-025-03043-1)
Supplement: Supplementary file 1 — Supplementary Material 1 [file 12875_2025_3043_MOESM1_ESM.pdf]

Supplementary Materials

Appendix A: 2018 Interview Guide

1. Please tell me about your practice setting and your role in the past 12 months.
    a. What are the areas of focus for your clinical practice?
2. Can you please tell me what you know about medical abortion and the new abortion pill called mifepristone?
3. What do you feel are the advantages of the new abortion pill?
    a. Do you see there being any downsides to having the abortion pill available in Canada?
4. Have you ever provided abortion care before? Can you please describe the care you provided?
    a. How do you feel about abortion?
5. Are you aware of the Society of Obstetricians and Gynaecologists of Canada mifepristone training program for physicians and pharmacists?
    a. If YES: Tell me your thoughts about it.
    b. If NO, explain what the training is before following up: Would taking this training be useful for you? (Why / why not?)
6. Can you describe the abortion care available in your practice setting or community?
7. How do other providers in your community feel about the abortion pill?
    a. What are your relationships like with other key people in your community who would be involved in providing the abortion pill? (Such as other physicians, pharmacists, managers)
    b. What are your relationships like with abortion providers?
    c. What relationships or networks do you feel are necessary for you to provide the abortion pill?
    d. Do you have a formal plan for implementing the abortion pill in your community? Do you know of anyone else's plans? What does they look like?
8. Are there any key individuals that have been leaders in implementing the abortion pill, either in your community or elsewhere in Canada? Can you describe what they did?
    a. Is there any person or organization you would describe as unsupportive? (What did they do?)
9. Have you exchanged information with anyone about the abortion pill, either inside or outside of your setting?
    a. What did that look like? (For instance, have you spoken to the media or contacted your college registrar?)
    b. Have you learned about any changes to abortion pill regulations or coverage? What have you learned? Where did you get the information?
10. I would like to talk a little more about any factors that may be an obstacle for you to providing the abortion pill. Has _______ been a factor? (How?)
    a. Cost (such as provincial coverage, financial disincentives, uncertainty about coverage)
    b. Billing codes (such as lack of billing codes; lack of compensation)
    c. Clinical workflow (such as counselling; following up; changing from a surgical to a medical abortion clinic; time pressure)
    d. Documentation (such as Health Canada forms, consent forms)
    e. Drug availability and dispensing (such as ordering it)
    f. Government support (such as political factors)
    g. Regulations (such as physician dispensing)

   h. Community presence of anti-choice attitudes (such as among protestors or colleagues)
   i. Having access to surgery, ultrasound, or labs
   j. Human resources (such as counsellors; staff burn out)
   k. Availability of information (such as confusion about regulations; where to get training; where to get updates)
   l. Training (such as the requirement to get training)
11. Would you ever consider providing medical abortion with mifepristone?
    a. If YES: What changes would make it easier for you to provide?
       i. Probe for changes to their personal opinions; professional support; training; policies and regulations; practical aspects of practice
    b. If NO: Why?
12. What support or feedback would be necessary for you to practice mifepristone medical abortion?
13. Are you aware of the Canadian Abortion Providers Support platform, also known as the "CAPS" website?
    a. If YES: Tell me your thoughts about it.
    b. If NO, explain what the website is before following up: Would joining this website be useful for you? (Why / why not?) / To help us make it useful for you, what information would you want from the website?
14. How will you know that the abortion pill is well received and used in your province?

I have come to the end of my questions.

1. Is there anything else you think I should know?
2. Do you have any questions for me?
3. Is there anyone else you recommend we talk to?

Appendix B: 2023 Interview Guide

1. Before we start our conversation, I have a few demographic questions. If you don't want to answer a particular question, we can skip it.

- What are the first three digits of your postal code?
- What is your discipline and/or specialty?
- What is your age?
- What is your current gender identity?
- Do you have experience providing abortion care?
- Are you currently offering medical abortion with mifepristone in your practice?

2. What motivated you to participate in this study?

3a. *For people who offer medical abortion:* Please tell me more about your experience providing mifepristone medical abortion?

3b. *For people who do not offer medical abortion:* What would motivate you to provide mifepristone?

4. I'm going to ask you about some of the different factors that may have impacted your intention or ability to offer medical abortion care. How has/have your _____ influenced your decision to provide/not provide medical abortions?

- Personal beliefs and values
- Clinical environment
- Colleagues
- Patient population and their needs
- Training
- Costs, coverage, and remuneration
- Regulations
- Pharmacy stock, location, or relationships
- Real or perceived stigma or harassment

5. Have you experienced challenges in supporting patients to access a mifepristone medical abortion? *Probe for the factors listed in Q4*

If no experience or intention to provide abortions jump to Q10

6. Can you please share the story of your first medical abortion case or cases?

7. Please describe for me any additional steps you took in becoming a medical abortion provider. *Probe for information, access to mentors, training.*

- Was anything confusing at first? (e.g., changes in restrictions, etc.)
- What was most helpful to you in getting started?
- What was surprising or challenging in getting started?
- What would you tell your past self about this experience that would have been useful to know?
- Now, what helps to sustain your ability to provide medical abortion?

9. How do patients know to approach you for managing an unplanned pregnancy? What are some things you have done to let the community know that you offer abortion care?

10. What makes for a positive communication with a patient when they need a medical abortion? What makes for a negative communication? What would you like to tell patients that would improve patient/provider communication about abortion?

11. *Final questions:* Is there anyone else you recommend I talk to? Is there anything else you think I should know? Do you have any questions for me?
